# Live Your Religion

**Published:** 1888-08

**Authors:** 


### LIVE YOUR RELIGION.

He preacheth best who liveth best—
Men heed not words, but actions;
By this smooth stone 'tis fair to test
The zeal of blund'ring factions.

A godly deed weighs more than creed;
An armor fit for trials
Is forged alone by hands that speed
In righteous self-denials.

To win mankind, the heart first find
By acts of right and reason,
For words alone will never bind
Like good deeds done in season.

Not, as we say, on Sabbath day,
But as we practice weekly,
Will people rate us, though we may
Preach long, and wise, and meekly.

Wouldst thou preach well? Then live thou well—
Who'd lead the way to glory,
And best the Gospel tidings tell,
Should *live* the old, old story. —*Christian Observer.*

